# Supplementary material for: An algorithm to build synthetic temporal contact networks based on close-proximity interactions data
Source: PLoS Comput Biol. 2024 Jun 13;20(6):e1012227. doi: 10.1371/journal.pcbi.1012227 (PMC11207132; doi:10.1371/journal.pcbi.1012227)
Supplement: S1 Table — Values were estimated per day, with mean and standard deviation (sd) presented here. Transitivity is not shown for the patient-staff subgraph as triangles of contacts cannot occur in this network. (DOCX) [file pcbi.1012227.s001.docx]

**Supplementary Table 1: Summary of network characteristics for the observed total network, patient-patient subgraph, staff-staff subgraph, and patient-staff subgraph, separated by weekday or weekend.** Values were estimated per day, with mean and standard deviation (sd) presented here. Transitivity is not shown for the patient-staff subgraph as triangles of contacts cannot occur in this network.

|  | **Total** | | **Patient-patient** | | **Staff-staff** | | **Patient-staff** | |
| --- | --- | --- | --- | --- | --- | --- | --- | --- |
| **Day type** | **Weekday** | **Weekend** | **Weekday** | **Weekend** | **Weekday** | **Weekend** | **Weekday** | **Weekend** |
| **Degree (sd)** | 14.51 (2.75) | 9.20 (2.16) | 5.87 (1.77) | 3.70 (1.04) | 6.67 (1.44) | 3.68 (0.80) | 8.85 (1.54) | 6.17 (1.19) |
| **Global efficiency (sd)** | 0.42 (0.02) | 0.34 (0.05) | 0.28 (0.06) | 0.16 (0.08) | 0.36 (0.05) | 0.20 (0.08) | 0.34 (0.01) | 0.25 (0.05) |
| **Density (sd)** | 0.08 (0.01) | 0.06 (0.01) | 0.05 (0.01) | 0.04 (0.00) | 0.09 (0.02) | 0.09 (0.01) | 0.05 (0.00) | 0.05 (0.01) |
| **Transitivity (sd)** | 0.37 (0.02) | 0.37 (0.02) | 0.40 (0.05) | 0.45 (0.05) | 0.53 (0.05) | 0.63 (0.08) | NA | NA |
| **Assortativity (sd)** |  |  |  |  |  |  |  |  |
| ***By degree*** | -0.10 (0.09) | -0.20 (0.09) | 0.22 (0.10) | 0.21 (0.12) | 0.13 (0.10) | 0.19 (0.22) | -0.36 (0.12) | -0.56 (0.08) |
| ***By ward*** | 0.55 (0.04) | 0.68 (0.04) | 0.73 (0.10) | 0.87 (0.06) | 0.68 (0.05) | 0.83 (0.06) | 0.42 (0.06) | 0.57 (0.07) |
