# Supplementary figures and images for: An algorithm to build synthetic temporal contact networks based on close-proximity interactions data
Source: PLoS Comput Biol. 2024 Jun 13;20(6):e1012227. doi: 10.1371/journal.pcbi.1012227 (PMC11207132; doi:10.1371/journal.pcbi.1012227)

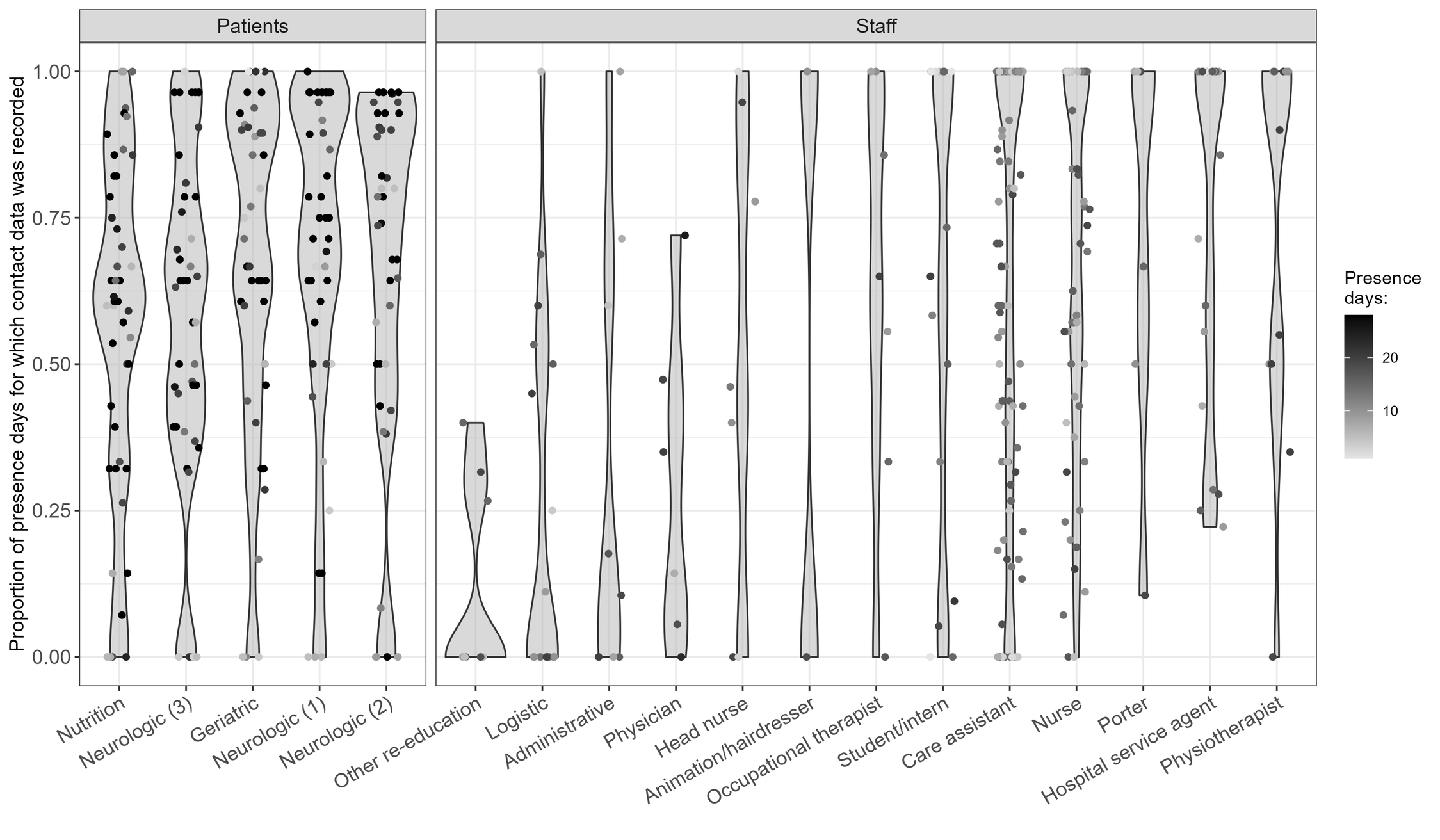

Supplement: S1 Fig — Each point is one individual’s proportion, calculated over the entire study period. The shading of the points indicate the number of presence days for each individual (darker = more presence days). (TIF) [file pcbi.1012227.s002.tif]

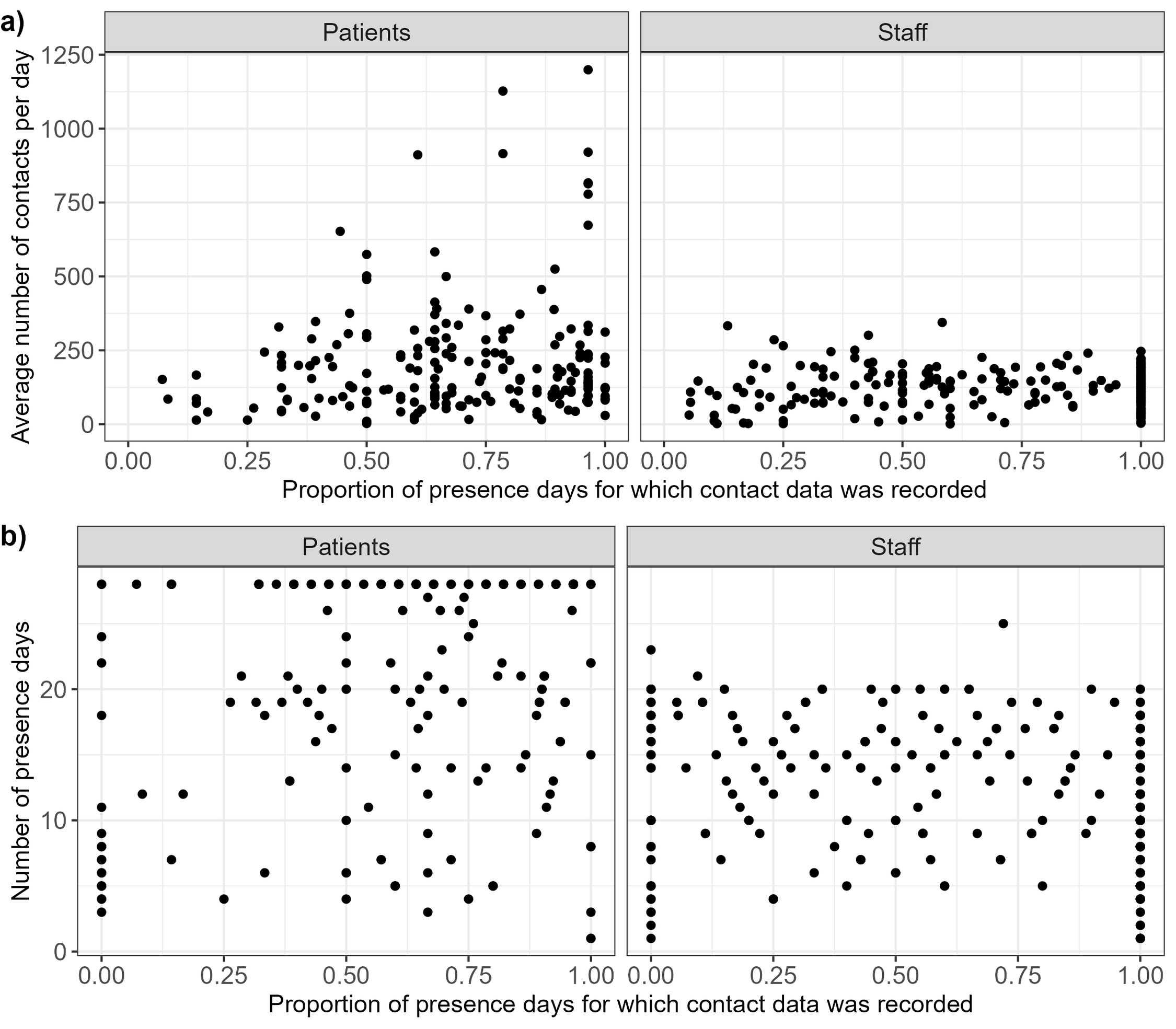

Supplement: S2 Fig — Each point is one individual. (TIF) [file pcbi.1012227.s003.tif]

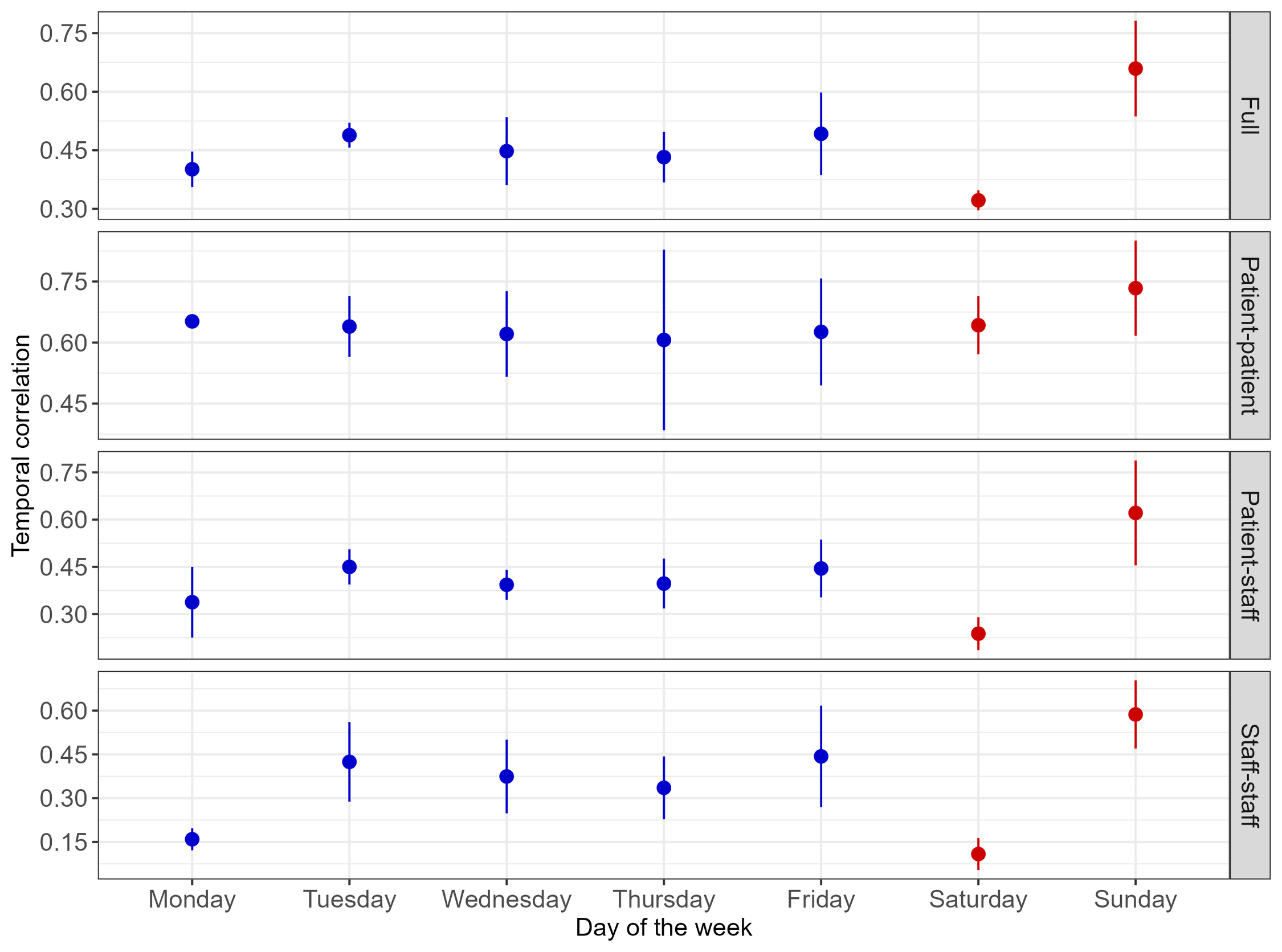

Supplement: S3 Fig — The correlation is calculated for each day by comparing it to the previous day; for example, the value on “Monday” indicates the correlation between the network on Monday and Sunday. Points indicate the mean correlation, and lines indicate the 95% confidence interval (1.96 times the standard deviation). Weekdays are shown in blue, and weekends in red. (TIF) [file pcbi.1012227.s004.tif]

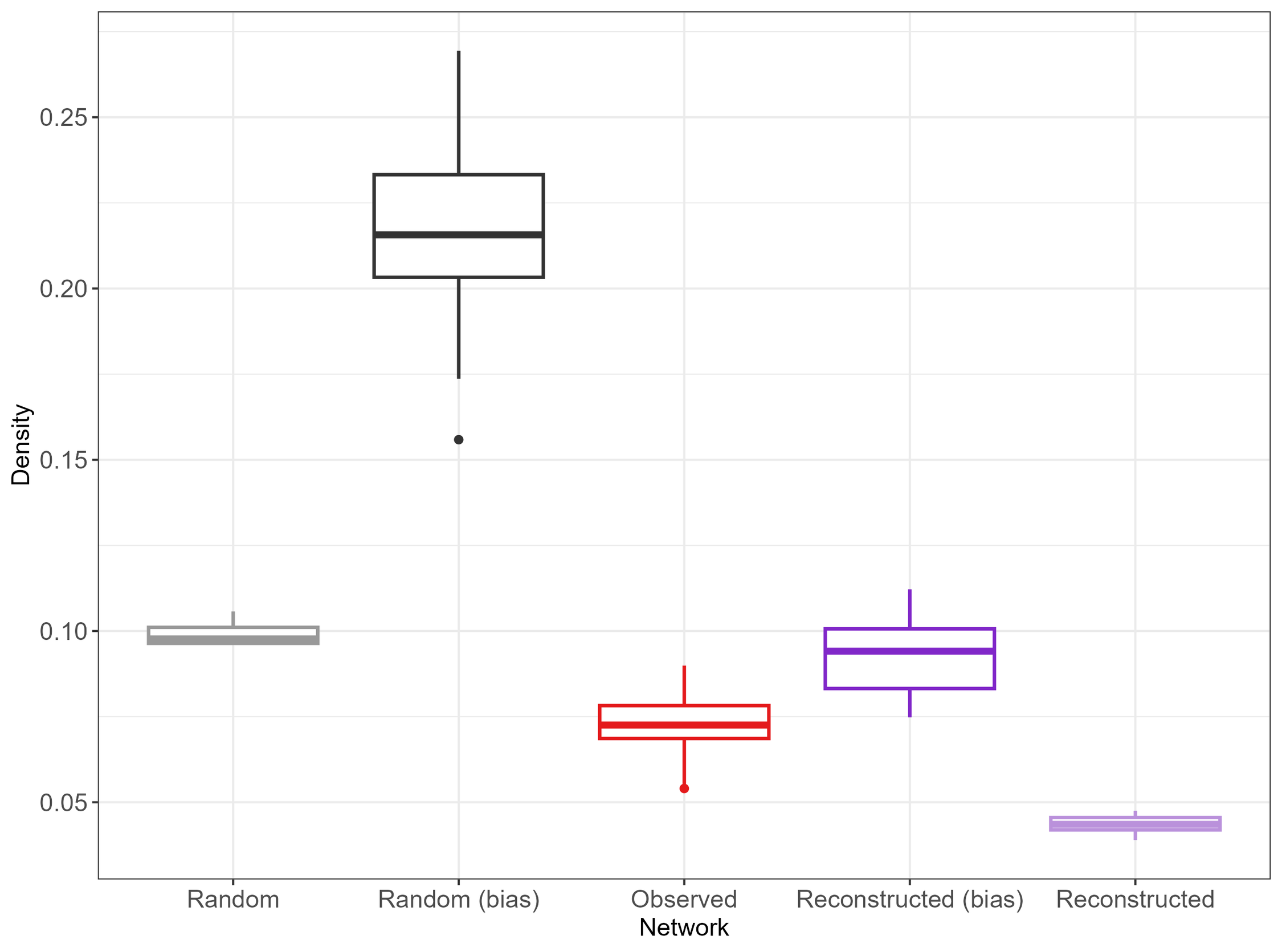

Supplement: S4 Fig — The reconstructed networks with observation bias exclude individuals from the network at times when they were known to not wear their sensors. The random networks did not take into account the ward-level structure of the contacts or the probability of recurring contacts. Boxplots for the observed network show the distribution of values calculated for each day. Boxplots for all reconstructed and random networks show the distribution of the median values calculated for each day across 100 networks. (TIF) [file pcbi.1012227.s005.tif]

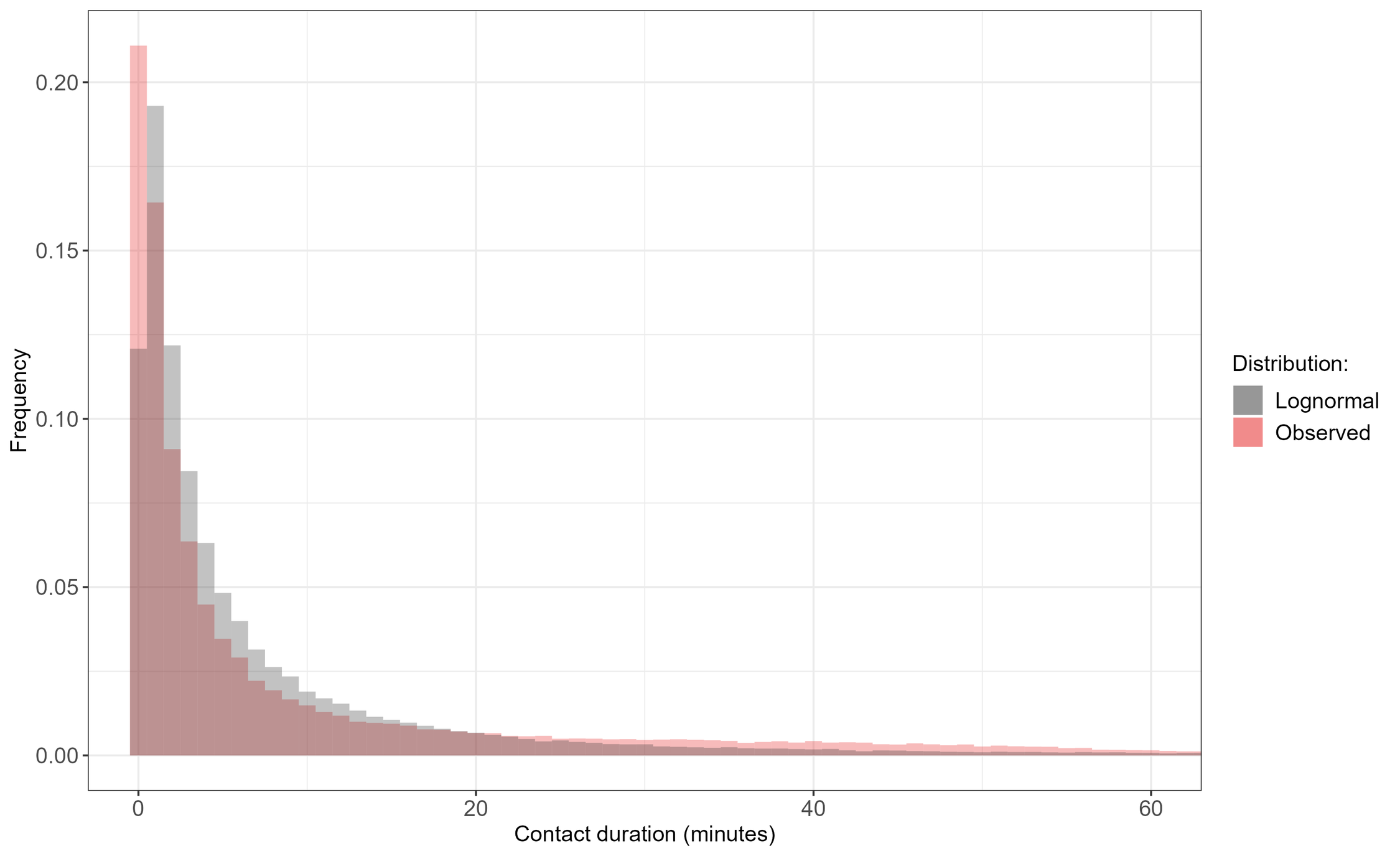

Supplement: S5 Fig — 100,000 samples are taken from a lognormal distribution is informed by the mean and variance estimated from the data. For ease of visualisation, the x-axis is truncated at 60 minutes. (TIF) [file pcbi.1012227.s006.tif]

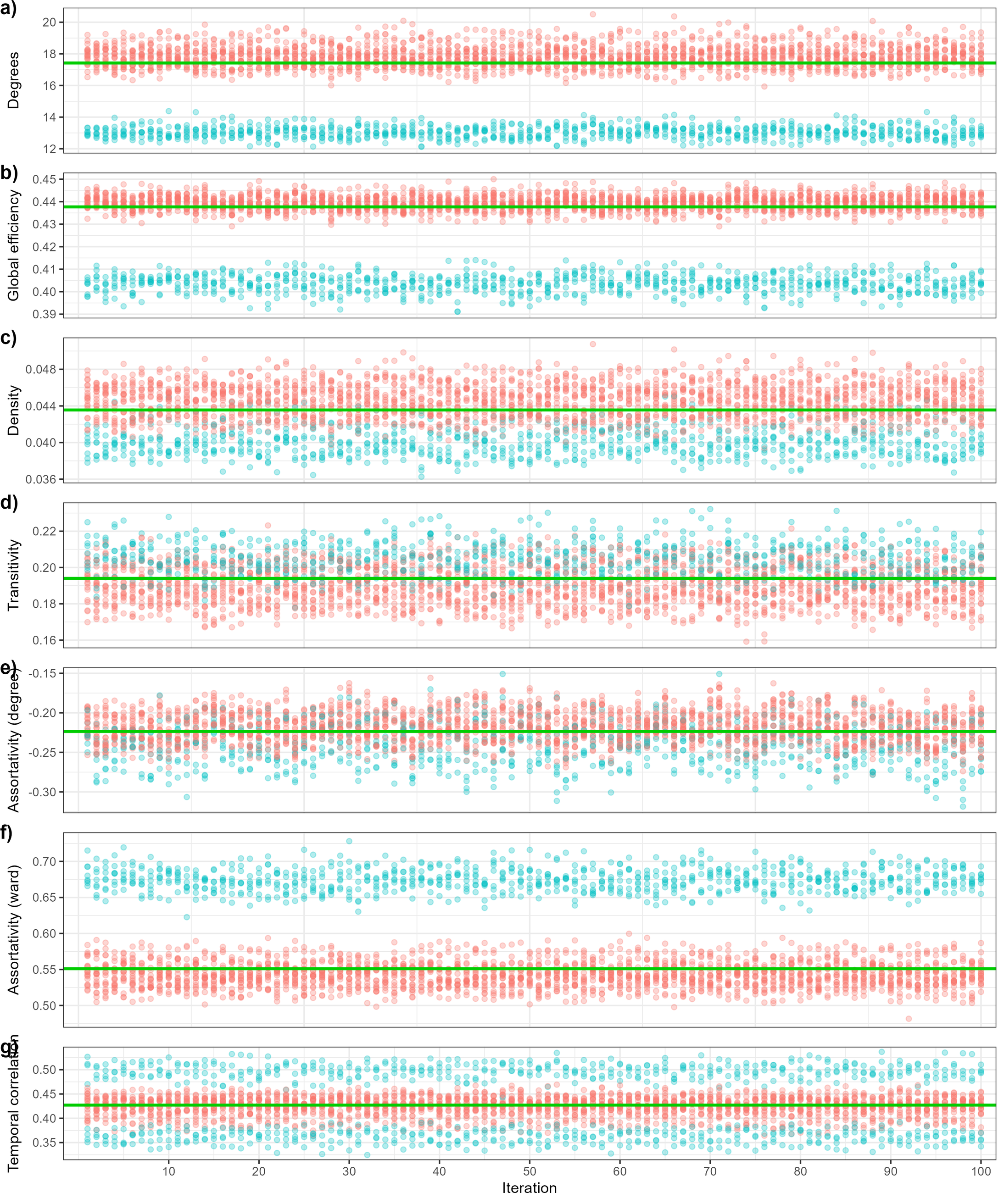

Supplement: S6 Fig — Here, 100 reconstructed networks without bias were generated independently, all informed by the same contact rates estimated from the i-Bird data. For each iteration, the distribution of the metric calculated for each day is shown. Red points are weekdays, and blue points are weekends. The green lines indicate the median value for the correspond metric across all networks and days. Only the distributions of assortativity by degree are significantly different between networks (Kruskal-Wallis test, p value < 0.001). (TIF) [file pcbi.1012227.s007.tif]

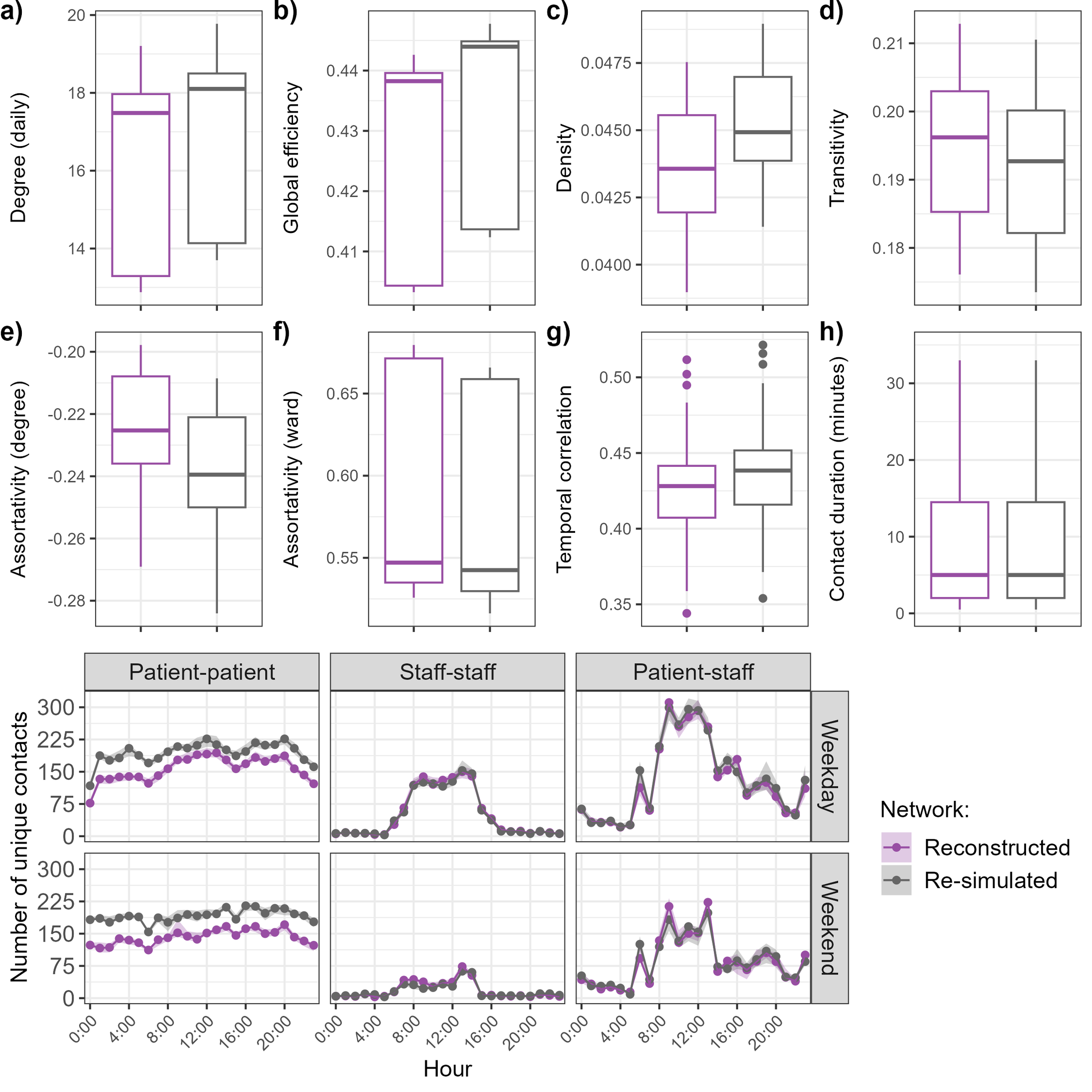

Supplement: S7 Fig — (TIF) [file pcbi.1012227.s008.tif]

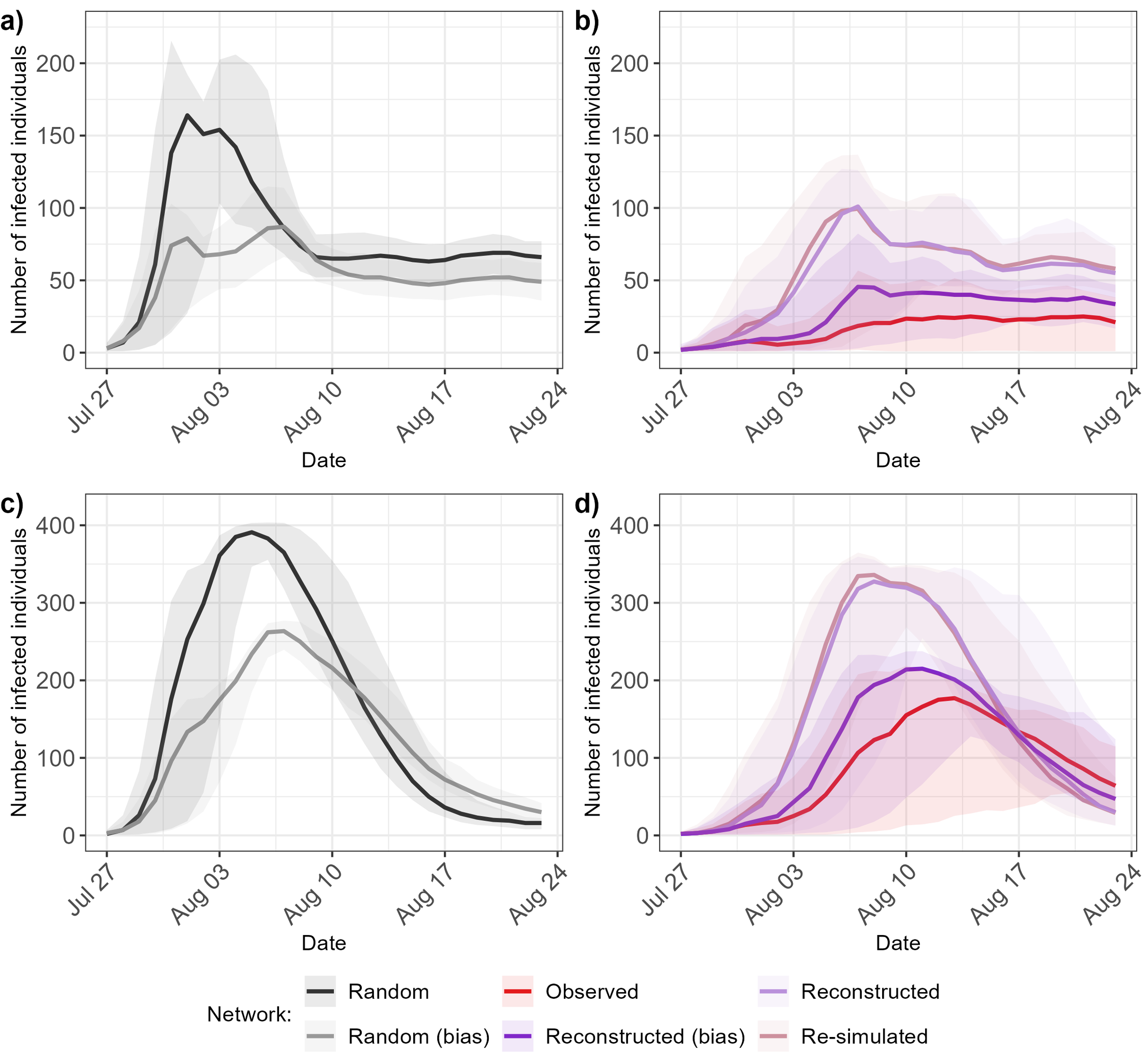

Supplement: S8 Fig — Comparison of resulting incidence dynamics depending on the networks, with an average duration of infectiousness of 2 days (a-b) or 10 days (c-d). a-c) Epidemic dynamics for the two random networks. Lines indicate median values, and the shaded areas indicate the interquartile range. b-d) Epidemic dynamics for the observed and reconstructed networks. Lines indicate median values, and the shaded areas indicate the interquartile range. (TIF) [file pcbi.1012227.s009.tif]

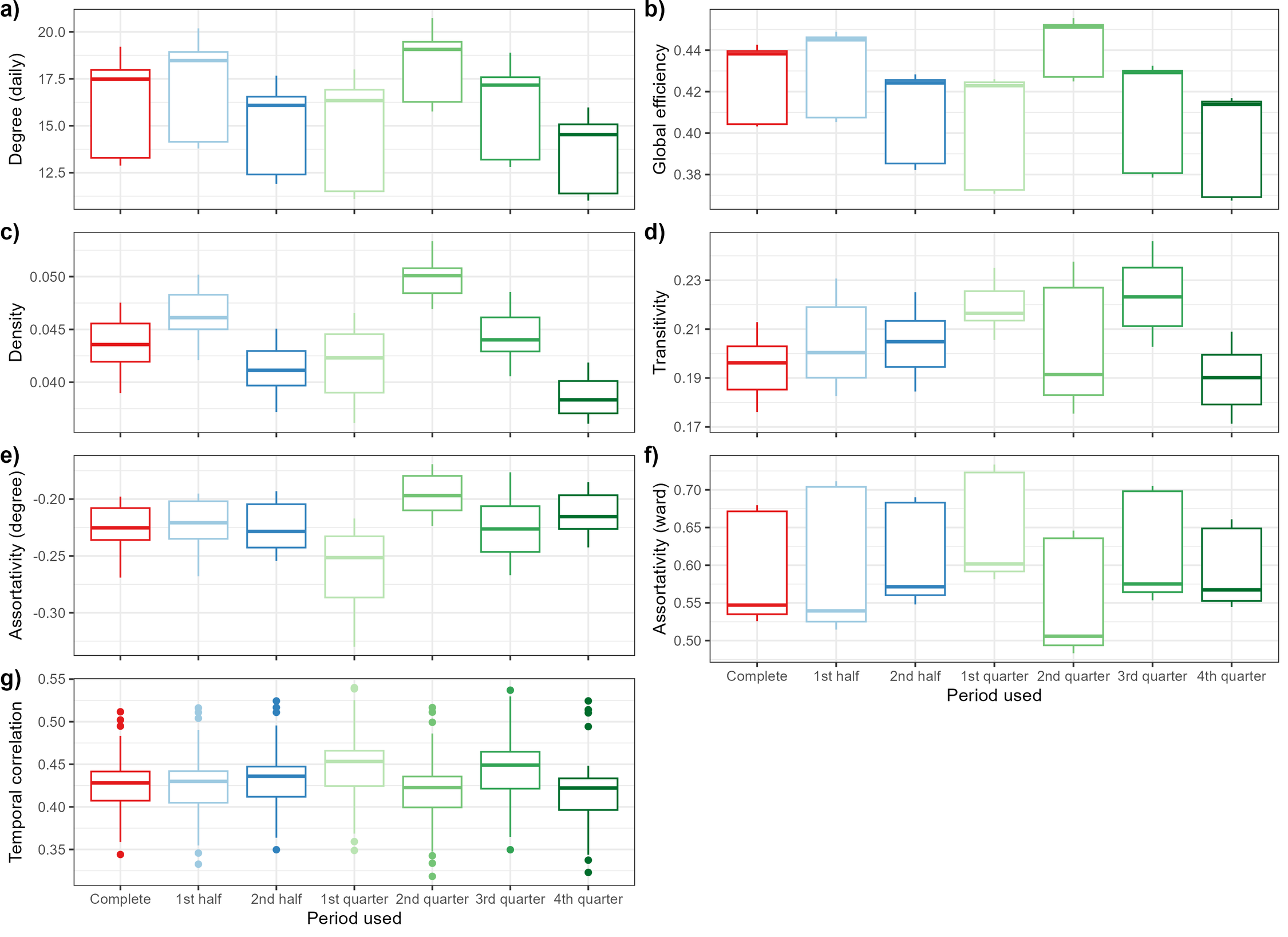

Supplement: S9 Fig — 1st quarter: 26/07–02/08, 2nd quarter: 03/08–09/08, 3rd quarter: 10/08–16/08, 4th quarter: 17/08–23/08. “1st half” includes the 1st and 2nd quarters, and “2nd half” includes the 3rd and 4th quarters. (TIF) [file pcbi.1012227.s010.tif]

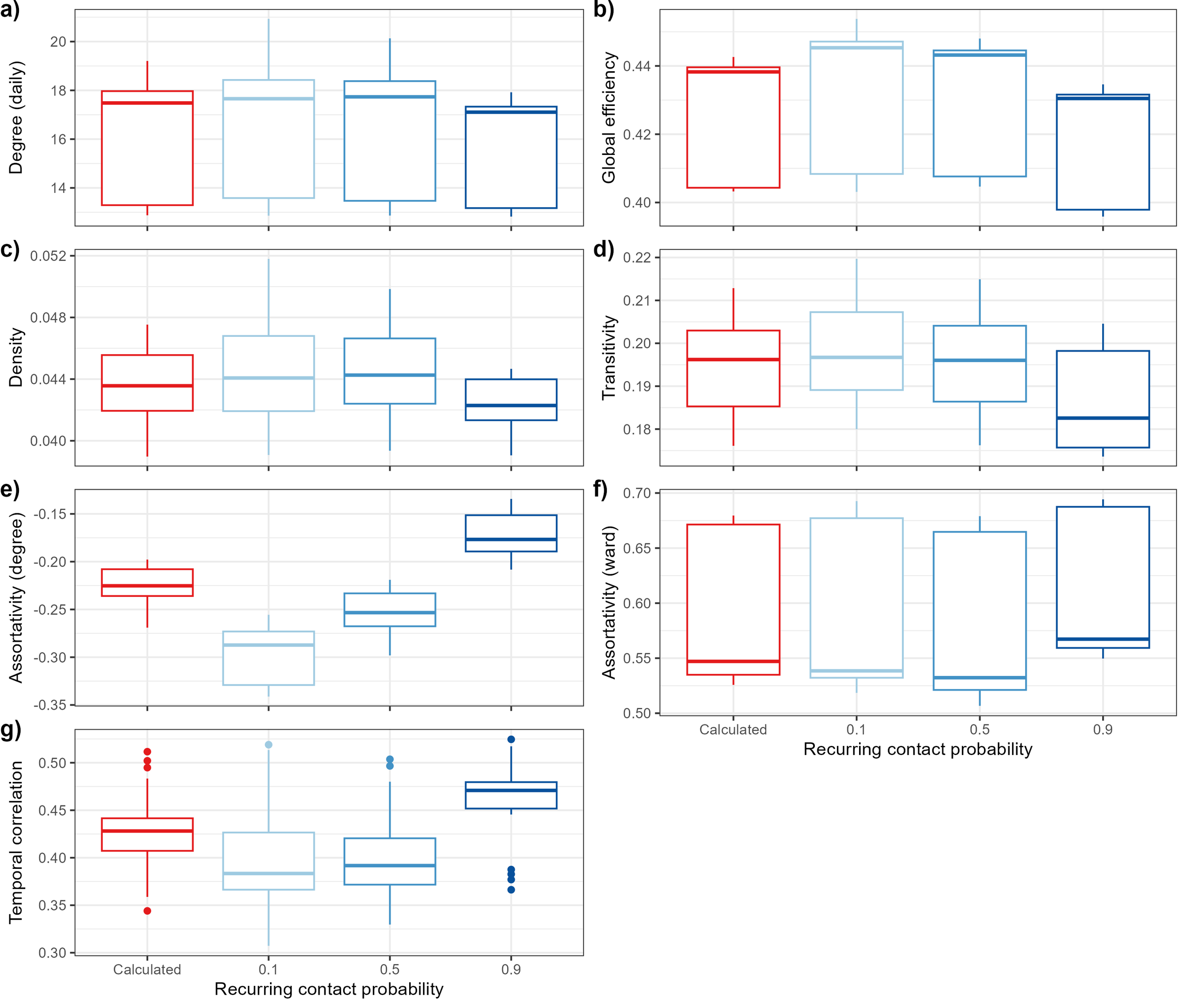

Supplement: S10 Fig — The calculated probabilities are 0.78 for patients and 0.71 for staff. In the other scenarios, the probabilities for patients and staff are set to the same value. (TIF) [file pcbi.1012227.s011.tif]
